# Male density, a signal for population self-regulation in *Alligator sinensis*

**DOI:** 10.1098/rspb.2019.0191

**Published:** 2019-04-10

**Authors:** Lan Zhao, Li-Ming Fang, Qiu-Hong Wan, Sheng-Guo Fang

**Affiliations:** 1 MOE Key Laboratory of Biosystems Homeostasis and Protection, State Conservation Center for Gene Resources of Endangered Wildlife, College of Life Sciences, Zhejiang University, Hangzhou 310058, People's Republic of China; 2 Changxing Chinese Alligator Nature Reserve, Changxing 313100, People's Republic of China

**Keywords:** male density dependence, population regulation, population dynamics, Chinese alligator, reptile, density stress

## Abstract

The regulation of population density is suggested to be indirect and occurs with a time-lag effect, as well as being female centred. Herein, we present a quantitative analysis on the precise, timely and male-dominated self-regulation of Chinese alligator (*Alligator sinensis*) populations. Analysis of 31 years of data revealed gender differences in regulation patterns. Population dynamics were restricted by male density rather than population density, and population growth was halted (birth rate = 0) when male density exceeded 83.14 individuals per hectare, until some males were removed, especially adult males. This rapid and accurate response supports the notions of intrinsic mechanisms and population-wide regulation response. Furthermore, density stress affected mating success rather than parental care to juveniles, i.e. females avoided unnecessary reproduction costs, which may represent an evolutionary advantage. Our findings highlighted the importance of further studies on related physiological mechanisms that focus on four characteristics: quantity breeds quality, gender differences, male density thresholds and nonlinearity.

## 1. Introduction

Density dependence is a general tendency and fundamental principle of population ecology [1]. It has been suggested that intrinsic (sociality and dispersal) and extrinsic (food and predators) factors interact to shape population cycles of vertebrates in nature [2]. Population cycles usually persist for years or even generations, and are subject to self-regulation with the death of offspring contributing the most to population dynamics [3]. This regulation represents fitness returns in which trade-offs exist between cost and benefit [4]. Parents, especially mothers, adjust to variable resource availability caused by density stress by altering sex ratio [5] and survival of juveniles [6,7]. In recent years, more studies have found that sex is a critical factor for understanding the effects of population density [8], and sex-specific response to density has been reported [9–11]. However, generational sex-specific effects have rarely been considered. A study on red squirrels (*Sciurus vulgaris L.*) suggested that reproduction rate decreased when female density was high [12], which suggests that females are at the core of population regulation. However, the importance of males in density-dependent population dynamics remains unclear.

In the present study, we quantitatively analysed the role of male-determined regulation of population dynamics in the Chinese alligator (*Alligator sinensis*). The Chinese alligator is a critically endangered species [13,14] that is endemic to China. Wild populations are only distributed in the Anhui Province, and the natural population size has remained at approximately 150 individuals for 20 years. To protect this species from extinction, two captive populations were established in Anhui Nature Reserve and Changxing Nature Reserve. However, both the Anhui and Changxing captive populations have encountered issues. At the Anhui Nature Reserve, 106 Chinese alligators were fed in a 330 m^2^ pool. They did not reproduce when the population density reached 3200 ha^−1^ but began reproducing again when the population density decreased to 88 ha^−1^, which was achieved by expanding the breeding area [15]. Similarly, we observed that the population in the Changxing Nature Reserve stopped reproducing when the population reached a specific density. Joanen & McNease [16] reported that the nesting success of the American alligator (*Alligator mississippiensis*), a relative of the Chinese alligator, was seriously affected by extrinsic factors, including flood, drought and predation. However, these factors could not explain the zero population growth in the two captive Chinese alligator populations because there was sufficient food, rarely floods and rarely any predators in the nature reserves.

Another feature of the observed zero population growth was that reproduction commenced when some individuals were removed from the populations. This indicated a real-time density-dependent regulation of population by factors other than intrinsic (sociality and dispersal) and extrinsic (predation and food) factors, which have been widely reported in other species. In the present study, we collected and analysed breeding data from the Changxing population to understand the self-regulation and population density dynamics of the Chinese alligator and to identify the key factors involved.

## 2. Methods

### (a) Study population

Different situations exist in the two populations of captive alligators in China. In the Anhui Nature Reserve, more than 10 000 alligators, including adults, were fed in cement ponds and were captured and transferred into greenhouses for overwintering. However, some of the captive individuals in the Anhui Nature Reserve and most individuals (except for hatchlings) in the Changxing Nature Reserve remain in the natural or restored habitats for natural breeding, cave digging and overwintering. We defined the captive populations which live in the natural or restored habitats as semi-natural populations because the conditions that they are exposed to are comparatively similar to those experienced by natural populations.

**Figure 1.**
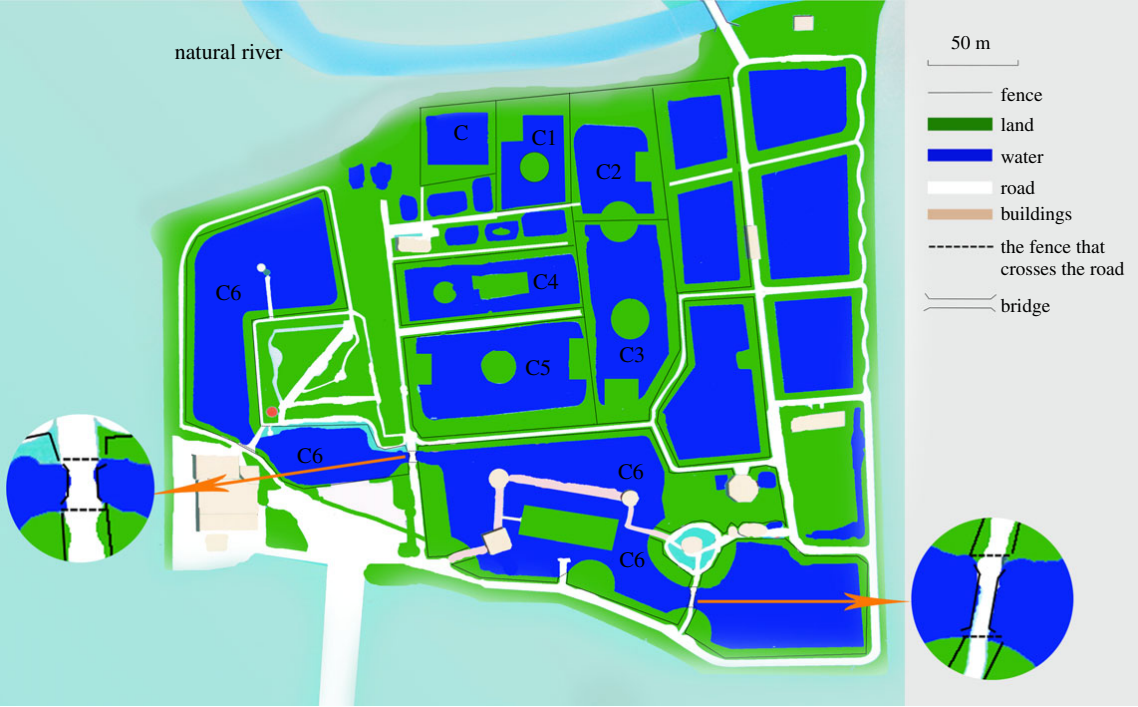
Organization of the pools for Chinese alligators (*A. sinensis*) in Changxing Nature Reserve.

The semi-natural population in the Changxing Nature Reserve (30°93′ N, 119°73′ E), located in the county of Changxing, Zhejiang Province, is herein referred to as the Changxing population. The Changxing reserve is an extension of the pristine habitat of the Chinese alligator. Area C, i.e. pristine habitat, covers an area of 0.54 ha and 11 adult alligators (three males and eight females) have been known to have naturally lived in this area since April 1979 and represent the founders of the Changxing population. In 1992, two adult males were captured from the wild to supplement the population in area C. The reserve has been building artificial habitats around the original wetland landscape since 1996 to accommodate the increasing population. The artificial habitats were numbered consecutively as C1, C2, C3, C4, C5 and C6. Each area comprises an independent pond and are cordoned off by fences or other barriers. The organization of the pools is shown in figure 1. Accordingly, the entire Changxing population was divided into seven subpopulations: C–C6. The year of establishment, area and number of founder individuals are listed in the electronic supplementary material, table S1.

### (b) Data collection

The breeding season of the Chinese alligator occurs in summer from mid-July to mid-September [17]. The staff at the Changxing nature reserve have been recording the population status in spring and the reproduction status in autumn every year since 1979. Females in the founding population first laid eggs in 1979. However, their preferred laying site was situated on the centre island of area C, and the island was submerged from 1979 to 1983 as a result of flooding. All nests were damaged, and thus, no data were recorded. The population parameters included the number of total individuals, males and females. The reproduction parameters included the number of nests, total eggs, incubated eggs, the juveniles that survived to winter, male juveniles and female juveniles. The methods related to number count, incubation, fertilized eggs identification, sex identification, age identification, and data selection have been listed in the electronic supplementary material, methods S1. For population data, see the electronic supplementary material, table S1; age structure of C and C1 area also see the electronic supplementary material, table S2.

### (c) Data analysis

We set the reproduction data per area per year as the samples, and 87 samples were considered in total. In addition to the basic data recorded by the staff of Changxing Nature Reserve, secondary parameters were also calculated. Density represents the number of individuals (total, males, females, nests, eggs and hatchlings) ha^−1^. The birth rate (here, a crude birth rate), usually the dominant factor in determining the population growth rate, was calculated using the total number of live births per 1000 births in a population yr^−1^ (see https://www.indexmundi.com/world/birth_rate.html). We used percentages because the population size of alligators in Changxing Nature Reserve is small. To evaluate the effect of population parameters on the birth process, the number of individuals in the mating season was considered. The formula used was

$$\mathrm{crude}\;\mathrm{birth}\;\mathrm{rate}=\frac{\mathrm{hatchlings}}{\mathrm{individual}\;\mathrm{numbers}\;\mathrm{in}\;\mathrm{mating}\;\mathrm{season}}.$$

SPSS software v. 20.0 was used for statistical analyses. Figures were drawn and data fitted using Origin 2017. The Shapiro–Wilk test was used to test for normality and to determine the distribution and expression of data. Values were represented as means ± standard deviations (*M* ± s.d.) and used to describe the central tendency and variation of the normally distributed data. The median (first quartile (*Q*1) and third quartile (*Q*3)) was used to describe the non-normal data. Pearson's correlation analysis was used to assess the degree of correlation between two variables; the data of which demonstrated a bivariate normal distribution. Variables that were not suitable for product–moment correlation analyses were subjected to a Spearman's correlation analysis. The degree of association between two random variables was measured by partial correlation to remove the effect of a set of controlling random variables [18]. The trend between two variables was depicted using a scatter diagram. Box-plots were used to present the characters of group data. The independent samples *t*-test was used to test the difference between the two groups. When the data of two groups were homoscedastic, a normal *t*-test was conducted; otherwise, an adjusted *t*-test was performed. Therefore, the degree of freedom was not uniform. Statistically significant differences were identified at the 95% level of confidence (*p* < 0.05) and the tests used were two-sided. The coefficient of variation (CV) was calculated to determine whether the data were discretized. The formula used was

$$\mathrm{CV}=\frac{s.d.}{M}\times 100.$$

## 3. Results

### (a) Growth mode of subpopulations

**Figure 2.**
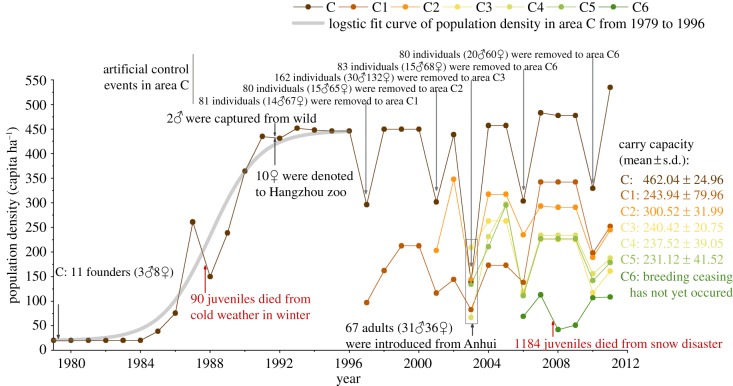
Population density dynamics of subpopulations of the Chinese alligator (*A. sinensis*). Grey arrowhead marks some immigration events of the whole population and the transfer events of area C. Red arrowhead marks deaths from 1979 to 2011. Carrying capacity of areas C–C5 is listed on the right.

As shown in figure 2, the size of each subpopulation initially increased, and then reproduction (i.e. population growth) decreased to zero. For instance, the population size of area C increased from 11 founders in 1979 to 244 individuals by 1993, and reproduction was thereafter halted for four consecutive years (figure 2; data are listed in the electronic supplementary material, table S1). The growth mode showed an *S*-type curve and the fitting formula of the logistic curve was

3.1 $$\begin{array}{r} y=\;446.76-\frac{446.76-20.36}{1+e^{-(x-1988)/1.4}};1980\leq x\leq 1996;\\ \;\mathrm{adj}.R^{2}=0.96,d.f.=15,p=0, \end{array}$$

where *y* represents the population density and the adjusted-fitness was 0.96, which indicated a good fit with the logistic model. The carrying capacity (i.e. equilibrium density) *K* was 446.76 capita ha^−1^.

The population in area C began to reproduce again when 81 individuals were moved to area C1 in 1996. In 1997, the first-generation offspring began to reproduce. However, reproduction was halted again when the number of individuals increased to 243 in 1998. A similar ‘start–stop–start–stop’ cycle was observed in areas C1–C5, and the birth rate reached zero when the number of individuals (i.e. population size) reached a certain level. The data of the subpopulations showed a strong population self-regulation mechanism.

The equilibrium density of area C was 462.04 ± 24.96 capita ha^−1^, while C5 exhibited the lowest equilibrium density with only 231.12 ± 41.52 capita ha^−1^. The different areas exhibited different equilibrium densities, which suggests that population density is a correlation factor rather than a decisive element. Therefore, the age structure and sex structure were evaluated to identify the trigger signal.

### (b) Trigger signal

#### (i) Sex structure

**Figure 3.**
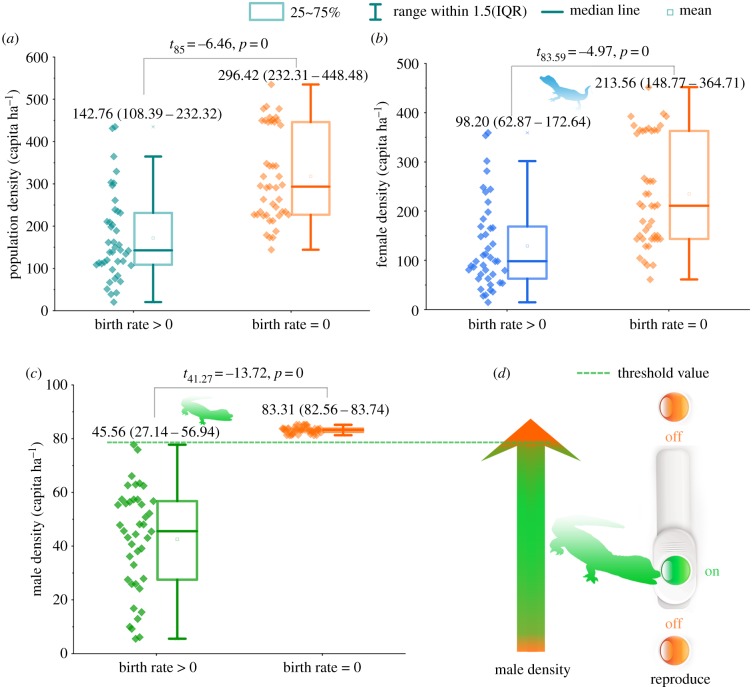
The difference of male density in the breeding and non-breeding samples. Variation in density of the population (*a*), females (*b*) and males (*c*) in the breeding and non-breeding samples. The lines above and below the bars represent the first (*Q*1) and third quartiles (*Q*3). The blocks within the bars represent the second quartiles (the median *Q*2). The fences were calculated using the following formulae: lower fence = *Q*1 – 1.5(IQR); upper fence = *Q*1 + 1.5(IQR). In the formula, the IQR (inter-quartile range) = *Q*3 – *Q*1. The asterisks plotted above the whiskers represent outliers. Values above the boxes represent the sample size and the median (*Q*1–*Q*3). Density data points are plotted on the left of each box chart. Each point represents the density for each year in each area. (*d*) The reproduction stopped when male density exceeded threshold or decreased to 0, while it occurred in the intermediate male density.

Spearman's correlation analysis revealed that the birth rate declined with the increase in population density (*R* = −0.61, *n* = 87, *p* = 0), male density (*R* = −0.84, *n* = 87, *p* = 0) and female density (*R* = −0.51, *n* = 87, *p* = 0), but not in response to population sex ratio (*R* = −0.10, *n* = 87, *p* = 0.38). This indicated that the decision signal for reproduction might be attributed to the density of one gender rather than the sex ratio. The discreteness of datasets showed distinction in the three density parameters. The range of data overlapped widely between breeding and non-breeding groups for both population and female densities (figure 3*a*,*b*). Male density was an exception. When the population entered into the non-breeding state, the male density was higher than that during the breeding state (figure 3*c*). The minimal value of the non-breeding group (81.23) was higher than the maximal value of the breeding group (77.76). Furthermore, the CV of the population density, female density and male density in the non-breeding state were 33.87%, 45.72% and 1.36%, respectively. This suggests that the male density (83.16 ± 1.13) was a constant value when the population stopped breeding and indicates that male density controls the self-regulation of the population and determines whether the population reproduces or not (figure 3*d*).

#### (ii) Age structure

**Figure 4.**
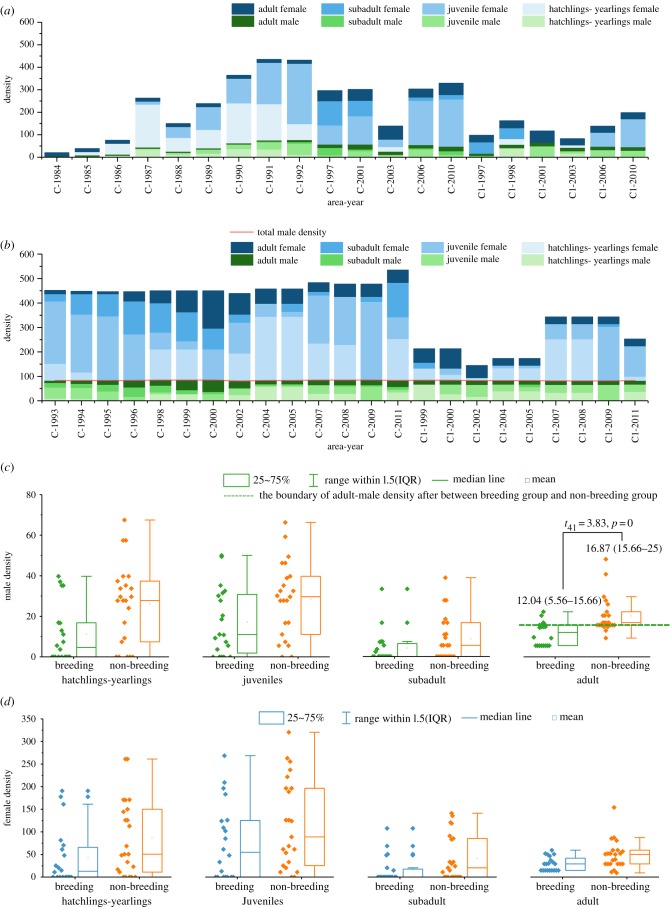
The sex–age structure of the Chinese alligator (*A. sinensis*) population in areas C and C1 in Changxing Nature Reserve. Age-density structure in the breeding samples (*a*) and non-breeding samples (*b*); variation in male density (*c*) and female density (*d*) at four age stages between the breeding and non-breeding samples.

The sex–age structure showed that the total male density (comprised hatchlings-yearlings, juveniles, subadults and adults) in the non-breeding sample group approached a constant value (figure 4*b*), but this was not apparent in the breeding group (figure 4*a*). Among the four age stages, male density and female density overlap between the breeding group and the non-breeding group, except for adult males (figure 4*c*,*d*). The adult-male density in the non-breeding group was significantly higher than that in the breeding group (*t*_40_ = 3.83, *p* = 0); however, the boundary is not as clear as it is with total male density (figure 4*c*). There is still 25% data overlap in the two groups, which indicates that there is a breeding response to adult-male density but that other factors also illicit such a response.

Further investigation in specific years has been considered. According to the data records presented in the electronic supplementary material, table S1, 81 individuals (14 males and 67 females), born in 1984, 1985, 1986 and 1987, were moved to area C1 in 1997. The ages of these individuals ranged from 9 to 13 years. The removed alligators were mostly adults and near-mature individuals. By 1996, the female offspring born in 1984 had matured; however, no reproduction occurred until they were removed to area C1. Reproduction in area C, which had been halted for 4 years, recovered after these individuals were removed. This suggested that excessive density stress hindered the increase in population density. Furthermore, the increase in juveniles restrained reproduction in the population.

All founder-males and founder-females in area C1 were mature in 1999 and 2000, respectively, and their offspring reached maturity at least 7 years later (i.e. from 2007 onwards). However, the non-reproducing phenomenon of area C1 occurred in 1999, 2000, 2004, 2005 and 2007. Specifically, 80 offspring were moved from area C1 to area C2 in 2000. These 80 individuals were born in 1998 and 1999 and they ranged in age from 2 to 3 years. After removing these young individuals, the reproduction in area C1 recovered in 2001. In 2002, 64 individuals were moved to C4. These 64 individuals comprised all of the offspring and 25 founders; thus, only adults remained in area C1. In 2003, these adults reproduced and gave birth to 77 surviving hatchlings (33 males and 44 females). In 2004, the reproduction stopped when these hatchlings reached seven months of age. Reproduction recovered when some alligators were removed (mainly adults and subadults) and soon halted when new hatchlings were supplemented into the population. This phenomenon was observed not only in area C1 but also in areas C2–C5. These findings indicate that the density of juveniles also contributes to density pressure in Chinese alligator populations. However, this effect is based on the adult-male density, which contributes the largest difference to breeding or non-breeding status among the four age stages.

### (c) Regulation mode

**Figure 5.**
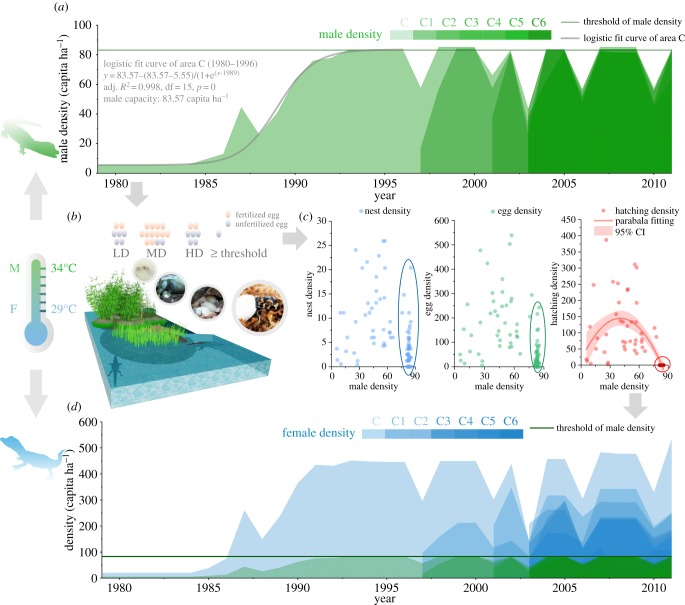
The mode of population self-regulation in the Chinese alligator (*A. sinensis*). (*a*) Male density dynamics of subpopulations. Thick line shows the growth curve of male density from 1979 to 1996 in area C; thin solid line shows male density threshold. (*b*) The hatching responses of alligators to different male densities in a certain habitat. LD, low density of males; MD, medium density of males; HD, high density of males. (*c*) The effect of male density on nest density, egg density and hatching density. The ellipses represent nest density (blue), egg density (green) and hatching density (red) when breeding ceased. The pink line indicates the fitting curve of the hatching and male densities, while the light pink area shows the 95% confidence interval (CI). (*d*) Population dynamics (involving female and male densities). The dynamic of female density and total population density had no fixation threshold value.

The dynamics of male density indicated that regular reproduction occurred when the male density was below the threshold value (figure 5*a*). There are four steps of reproduction in each breeding season: mating, ovulation, egg-laying and incubation (figure 5*b*). The adult females still built nests and laid eggs despite not producing any hatchlings during the non-breeding years. However, the nest density (independent sample *t*-test: *t*_60.828_ = −5.534, *p* = 0) and egg density (independent sample *t*-test: *t*_52.833_ = −6.674, *p* = 0) were significantly lower in the non-breeding group than in the breeding group. According to the calculated age structure data (only for areas C and C1), the ratio of nests to adult females in the non-breeding group was also lower than that in the breeding group (Mann–Whitney test: *Z* = −4.369, *n* = 43, *p* = 0). This may indicate that ovulation may be induced or impeded by the males because the ratio of adult females that took part in the breeding activities responded to male density (*R* = −0.561, d.f. = 43, *p* = 0), especially adult-male density (*R* = −0.677, d.f. = 43, *p* = 0).

In addition, nest density ranged from 0 to 20.36 at the threshold of male density, while the egg density aggregated to zero thereafter, and lastly the hatchling density reached zero (figure 5*c*). In addition, the fertility rates of the non-breeding group were all zero. Therefore, the ovulation ratio and fertility ratio responded to density stress. This indicated that density stress acts on the mating success of adult individuals and is different from the parental care to juveniles, which has been reported as the main intrinsic factor in other species [5–7].

Male density showed similar parabola trends as nest density, egg density and hatchling density (figure 5*c*). We used the hatchling density to investigate the effect of male density on mating success. A parabola function explained more about the relationship between male and hatched juvenile densities than a monotonic linear curve (figure 5*c*). The fitting formula used was

3.2 $$\begin{array}{rl} y= & -0.08{\left(x-40.48\right)}^{2}+144.83;0\leq x\leq 81;\\ & \mathrm{adj}.R^{2}=0.49,d.f.=84,p<0.01, \end{array}$$

where *x* is male density and *y* is the corresponding hatchling density. The goodness of fit was only 0.49, which indicated that this fitting model was not suitable to conduct quantitative inference. This may have been because the incubation success was also affected by the incubation conditions, especially incubation temperature. Although the nest site and nest parameters, such as nest materials, can be chosen by the mother alligator, the climate is uncontrollable. However, the qualitative trend of the fitting model was reasonable because the *p*-value was less than the set significance level. With the increase in male density, the density of hatchlings increased at first to a maximum value of 40.5 ha^−1^, and then decreased with further increases in male density. The density of hatchlings decreased to zero when the male density reached 81.23 capita ha^−1^. There was an optimal range of male density that enabled high breeding success to be maintained. Extremely high or low male densities are not conducive to the development of a population. By controlling the hatching success, male density affects the female density dynamic. In species with a constant sex ratio, population density is proportional to male density. In species that have environmentally determined sex, such as the Chinese alligator, the female density and population density do not change proportionally with male density because the sex ratio varies with environmental variation (figure 5*d*). This phenomenon may have contributed to the lack of pattern in the population density that we initially observed (figure 2).

## 4. Discussion

The population dynamics of the Chinese alligator observed in this study were consistent with those of the saltwater crocodile (*Crocodylus porosus*). A report on 12 wild subpopulations (1975–2009) of saltwater crocodiles (*C. porosus*) revealed two important phenomena: (i) the number of crocodiles did not increase much, although their average body size continued to increase, which indicates that density stress is related to the increase in density rather than the increase in biomass; and (ii) the expected carrying capacity of different subpopulations showed considerable variation in abundance and biomass [19]. The results of our study are consistent with this previous study, i.e. that the increase in the number of individuals contributed to density stress, and also that the different subpopulations showed variation in capacity. The lack of information available to the authors of the previous study, however, made it impossible to identify the reasons why the different rivers supported different densities of crocodiles. Therefore, the researchers indicated that it may have been a result of differences in habitat quality along the river systems.

In Chinese alligators, the analysis of data spanning 31 years provided us the opportunity to elucidate the decisive role of male density in the regulation of the population of this species. The population-density dependence or different carrying capacities are a misinterpretation of male-density dependence. The same driving force, i.e. male density dependence, may also provide a reasonable explanation for the variation in carrying capacity of different subpopulations of crocodiles (*C. porosus*). However, detailed sex-structured data are needed to confirm this.

Another previous study reported that the removal of large male caimans (greater than 180 cm total length) reportedly resulted in a population increase [20]. This supports a compensatory mechanism that would be triggered by the removal of large males, i.e. recruitment and the enhanced growth to maturity of subadult males. In addition, high adult male, but not female, density has been found to reduce juvenile survival owing to the predation of adult males on juveniles in a territorial lizard (*Anolis sagrei*) [21]. According to the data pertaining to Chinese alligators, another mode may be involved, which differs from such compensatory mechanisms and predation effect. The removal of male individuals, especially adult males, caused disinhibition of reproduction rather than enhancing the survival of juveniles and subadult males. An advantage of this birth-inhibition regulation mode is the reduced cost of reproduction, so that the adult females conserve energy and can reproduce in the next breeding season once the density stress reduces. However, this does not mean that density stress has no impact on the survival rate of juveniles and subadults of the Chinese alligator. The alligators in Changxing Nature Reserve had sufficient food and were protected from predators. The hatchlings were raised by breeders, and therefore, their death rate was independent from the population density stress. Scarcely any deaths occurred after the Changxing alligators reached 2 years of age. In wild populations, the density stress may also affect the age-structured death process. However, because other factors that are important in the wild were unconsciously controlled in the Changxing nature reserve, the sex-specific and real-time regulation of the birth process could be detected. The three cases outlined above for three different species indicate that male density effects both reproduction and survival, and may contribute towards a general regulation mode in reptiles.

The dynamics of density pressure in Chinese alligators are extremely rapid. This provides ecological evidence for the role of hormone secretions in population regulation. Christian [22] proposed that stress, as a factor that stimulates the adreno-pituitary system, results in the secretion of the adrenocorticotropic hormone in order to release hydrocortisone. Elsey *et al*. [23] found that the level of plasma corticosterone was positively correlated with the stocking density of both captive adults and juveniles of the American alligator, which is a relative of the Chinese alligator; while the formation of density stress may refer to the physical (vision and bellow [24]) or chemical (pheromone [25]) communication. However, the physiological mechanisms require a comprehensive study and this study provides some advice. Our empirical data suggest that there are three aspects of population regulation: gender differences, nonlinear and quantity breeds quality. Some physiological studies [26–33] only proposed two or three levels: blank, high density and low density. These conclusions are, however, not complete for the following reasons: (i) stress might be a response to the density of one gender rather than population density; (ii) the responses of individuals and the overall population to stress are not linear; both high and low densities may have equal impacts on a population which will thus lead to a one-sided conclusion or conflicting viewpoints; and (iii) it is difficult to guarantee high density in a true high-density range and vice versa because the change in quantity does not cause an essential change within a certain range. Therefore, we recommend that future studies on physiological mechanisms also consider sexual differences and gradient levels.

**Figure 6.**
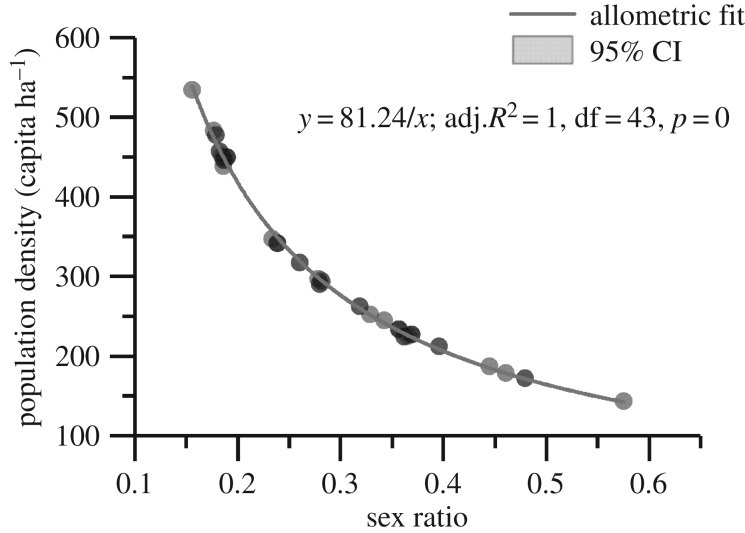
Allometric function between the population density and sex ratio of the non-breeding group in the Chinese alligator (*A. sinensis*) population in Changxing Nature Reserve. The sex ratio was calculated from the proportion of males. The grey line indicates the fitting curve of population density and sex ratio in the non-breeding group, while the light grey area shows the 95% confidence interval (CI). The 95% CI is going to overlap with the fitting curve as successful fitting.

Another detected effect of male density was the negative relationship between the population density and the sex ratio (*R* = −0.498, *n* = 87, *p* = 0). In the non-breeding group, an inverse-proportion function fitted well the two parameters (figure 6) when male density was a constant value. The sex ratio and population density were linked to male density. This furthered our understanding of the population dynamics and evolution of the sex ratio in this species. The female-biased sex ratio (1 : 4.517) [34] of the Chinese alligator can help to maintain the male density below the threshold value to ensure that sustainable reproduction takes place in the population.

The male density-dependent pattern is important for the effective management and protection of Chinese alligators, an endangered species. The natural population of the Chinese alligator is fragmented in several habitats. Our findings showed that reproduction will be halted if the male, especially adult-male density is excessive, except for some male alligators that disperse to other habitats. However, if habitat fragmentation is extensive, it would not benefit the population to continue increasing as it would be difficult for the male alligators to disperse to a new habitat. Interconnection of the fragmented habitats can alleviate this issue and allow the alligators to disperse from populations with high-density stress to those with low-density stress. It is recommended that if male density is maintained at approximately 40.5 ha^−1^, the population will achieve maximal reproduction success. In addition to male density, the sex ratio, which is affected by climate and intrinsic factors, also plays an essential role in the development of the population. A system analysis regarding the sex ratio of Chinese alligators should be conducted in a future study.

## Supplementary Material

The detailed methods of data collection and history data of Changxing population

## Data accessibility

The datasets supporting this article have been uploaded as part of the electronic supplementary material.
