# Supplementary material for: Male density, a signal for population self-regulation in Alligator sinensis
Source: Proc Biol Sci. 2019 Apr 10;286(1900):20190191. doi: 10.1098/rspb.2019.0191 (PMC6501674; doi:10.1098/rspb.2019.0191)
Supplement: The detailed methods of data collection and history data of Changxing population [file rspb20190191supp1.pdf]

Proceedings of the Royal Society B

The supplementary material for

Male density, a signal for population self-regulation in *Alligator sinensis*

DOI: 10.1098/rspb. 2019.0191

Lan ZHAO[1], Li-Ming FANG[2], Qiu-Hong WAN[1], & Sheng-Guo Fang[1, *]

[1] MOE Key Laboratory of Biosystems Homeostasis & Protection, State Conservation Center for Gene Resources of Endangered Wildlife, College of Life Sciences, Zhejiang University, Hangzhou 310058, China

[2] Changxing Chinese Alligator Nature Reserve, Changxing 313100, China

Correspondence and requests for materials should be addressed to

sgfanglab@zju.edu.cn

Supplementary methods S1

Number count. Initially, the population size was small, and the staff could count the alligators during their daily feeding. The feeding season lasts from May to October every year. Once the population reached 200–300 individuals, counting in this way became difficult. The Chinese alligator has become a national-level protected animal; therefore, the nature reserve staff captured the alligators to confirm the number and sex every spring so that they can report the data to State Forestry Bureau of China.

Incubation. Eggs were collected every year from the alligator nests after 50 days of incubation, i.e. ~1 week before hatching. Eggs were incubated in artificial hatchery rooms. During the egg collection, the number of nests and the total number of eggs were recorded, and the number of hatchlings were record after hatching. The fertilisation status was examined in the nests in which all egg remained unhatched. Hatchlings were raised to survive the first winter and sexed.

Identification of fertilised eggs. Egg candlers were used to determine the fertilisation of eggs, whereby light was shone on the eggs in a dark environment. The opaque banding on the shell would be observed if the egg was fertilised [1], and infertile eggs would be translucent [2].

Sexual identification. The sex of adults and sub-adults was determined by examining the cloaca for the presence of a penis. In male adults, the penis was determined by touch by moving the finger from side to side along the ventral floor of the cloaca [3,

4]. The sex identification of hatchlings was conducted following the methods initially used for *Crocodylus johnstoni* and *C. porosus*. This involved inserting fine curved forceps into the cloaca and spreading them to view the clitero-penis. In females this was defined as “sharp” and “small”, while in males it was defined as “tubular” and “large” [5]. The sexing of hatchlings was usually conducted when they reached three months old. Before 1996, the hatchlings were released into a small part of a pond in the original habitat and protected, and the juveniles and the founders all coexisted in the same pond. It was difficult to distinguish the size of the clitero-penis in hatchlings raised to 4–5 years old in the original habitat (> 30 cm snout-vent length), i.e. to reconfirm their sex and then release back into the pool. After 1996, the sex of hatchlings was identified at 2–3 months old with reference to the identification methods used for *A. mississippiensis*. This method involved determining the colour of the clitero-penis, an important character for sexing alligators. The male clitero-penis is typically red, round, and large in comparison to the female organ, which is white, sharper, and smaller [6]. After sex identification, the alligators were released into the same pool as their mothers.

Age identification. Male and female Chinese alligators mature at 10 and 11 years old, respectively. Juveniles were mainly categorised by age. If the age was unclear, the body length (i.e. total length) was assessed and used to identify the age. The distinguishing standards used were as follows:

Hatchlings-yearlings: $\leqslant$ 2 years-old, body length $\leqslant$ 30 cm;

Juveniles: males = 3–6 years old or females = 3–7 years old, body length = 31–120 cm;

Sub-adult: males = 7–9 years old or females = 8–10 years old, body length = 121–150 cm;

Adult: males ⩾ 10 years old; females ⩾ 11 years old, body length ⩾ 151 cm.

Originally, the age structure data were not recorded each year for each area. However, the selection criteria of individuals removed to the new areas was explicit, and the number and age of dead individuals were also recorded. The selective order of founders in the C1–C6 area was: large offspring > small offspring > founders of the original area. In each age group, half of the offspring were left behind in the original habitat and the other half were moved to the new area in age order, i.e. from old to young. If more than half the individuals were removed, then the older offspring were selected first and then the younger offspring. The founders in the original area were considered last. The age structure of areas C and C1 were calculated for every year and the data are presented in Table S2. However, this age structure is not precise. It was difficult to determine the age structure in areas C2, C3, C4, C5, and C6 to a high degree of accuracy because they should be based on the age structure of areas C1 and C2. Therefore, the age structure data of areas C2–C6 are absent.

Data selection. A destructive snow and ice disaster occurred in 2008 which led to the death of 1184 alligators. To avoid another such disaster, the reserve changed its management protocol after 2011. In 2011, areas C–C6 were divided into 18 new

habitats in succession and all the hatchlings were moved to a greenhouse to overwinter, and then released into separate pools where they were protected during the following spring. These juveniles were moved to a reintroduction area when they were 6 years of age. Owing to this artificial control, we mainly analysed the data before 2011.

Supplementary references

1. Grahame J.W.W., Manolis S.C., Whitehead P.J., Dempsey K. 1987 The Possible Relationship between Embryo Orientation Opaque Banding and the Dehydration of Albumen in Crocodile Eggs. *Copeia* **1987**(1), 252-257. (doi:10.2307/1446070).

2. Chen B., Hua T., Wu X., Wang C. 2003 Research on the Chinese alligator (In Chinese). Shanghai Scientific and Technological Education, Shanghai, pp.312-313.

3. Bradt G.W. 1938 A study of beaver colonies in Michigan. *Journal of Mammalogy* **19**(2), 139-162. (doi:10.2307%2F1374609)

4. Chabreck R.H. 1963 Methods of capturing, marking and sexing alligators. In *Proceedings of the Annual Conference of the Southeastern Association of Game and Fish Commissioners*, pp. 47-50. (doi:10.1007/978-1-349-03711-7_2)

5. Webb G., Manolis S.C., Sack G.C. 1984 Technical Note: Cloacal Sexual of Hatchling Crocodiles. *Wildlife Research* **11**(1), 201-202. (doi:10.1071/WR9840201).

6. Allsteadt J., Lang J.W. 1995 Sexual Dimorphism in the Genital Morphology of Young American Alligators, Alligator mississippiensis. *Herpetologica* **51**(3), 314-325. (doi:10.1016/0162-3095(95)90001-2).

Supplementary table S1

The population events and breeding situation in each of the evaluated years.

“NC” means Not Clear for the data scarcity; ♀: female; ♂: male. The data in the table represent the number of individuals.

Area name	Year	Manual intervention events before breeding season		Population situation in breeding season			Breeding situation			Survival situation						Notes
		Emigration	Imigration	Total individual number	Male number	Female number	Nest number	Egg number	Hatchlings	Survive to 3 months-old			Deaths events (after 3 months-old)	Survival number to mature		
										Survival number	Male hatchings	Female hatchings		M	F	
	1979		1♂2♀are original inhabitants; 1♂2♀captured from Er-jie-ling; 2♀ captured from Guan-yin-qiao; 2♀captured from Xian-shan; 1♂captured from Si-an	11	3	8	NC	NC	0	0	0	0	0	0	0	The egg laid on the island, but the island was submerged by floods
	1980			11	3	8	NC	NC	0	0	0	0	0	0	0	
	1981			11	3	8	NC	NC	0	0	0	0	0	0	0	
	1982			11	3	8	NC	NC	0	0	0	0	0	0	0	
	1983			11	3	8	NC	NC	0	0	0	0	0	0	0	
	1984			11	3	8	2	27	10	10	2	8	0	2	7	
C	1985			21	5	16	6	83	64	52	9	43	1♀ born in 1984; 4♂9♀ born in 1985; 3♂15♀born in 1985 were preyed by the weasel	2	19	
	1986			41	7	34	6	138	134	101	17	84	0	6	29	
	1987			142	24	118	4	79	31	29	7	22	90 (11♂55♀ born in 1986, 6♂18♀born in 1987)	1	4	
	1988			81	14	67	5	98	51	48	8	40	0	8	40	
	1989			129	22	107	6	123	74	68	12	56	0	12	56	
	1990			197	34	163	5	94	43	38	7	31	0	6	9	
	1991			235	41	194	6	111	62	42	7	35	36 (3♂22♀ born in 1990; 5♂6♀born in 1991)	0	3	
	1992	10 ♀ were denoted to Hangzhou zoo	2 ♂ (Captured from wild on April 23 1992)	233	42	191	8	159	47	26	4	22	17 (1♂10♀ born in 1991; 6♀ born in 1992)	3	14	
	1993			244	45	199	6	117	0	0	0	0	0	0	0	

	1994			242	45	197	3	57	0	0	0	0	2♀ (Fight for nest site and died)	0	0
	1995			241	45	196	1	14	0	0	0	0	1♀ (Fight for nest site and died)	0	0
	1996			241	45	196	0	0	0	0	0	0	0	0	0
	1997	81 individuals (14♂67♀) were remove to C1		160	31	129	11	151	104	83	15	68	0	15	68
	1998			243	46	197	11	131	0	0	0	0	0	0	0
	1999			243	46	197	2	27	0	0	0	0	0	0	0
	2000			243	46	197	1	7	0	0	0	0	0	0	0
	2001	80 individuals (15♂65♀) were remove to C2		163	31	132	14	291	163	74	13	61	0	13	61
	2002			237	44	193	0	0	0	0	0	0	0	0	0
	2003	162 individuals (30♂132♀) were remove to C3	13 mature individuals (6♂7♀) were introduce from Anhui nature reserve	75	14	61	13	258	209	172	31	141	0	31	141
	2004			247	45	202	2	12	0	0	0	0	0	0	0
	2005			247	45	202	4	81	0	0	0	0	0	0	0
	2006	83 individuals (15♂68♀) were remove to C6		164	30	134	14	272	168	97	16	81	0	NC	NC
	2007			261	46	215	5	47	0	0	0	0	3♀ born in 2006	0	0
	2008			258	46	212	1	8	0	0	0	0	0	0	0
	2009			258	46	212	0	0	0	0	0	0	0	0	0
	2010	80 individuals (20♂60♀) were remove to C6		178	26	152	10	194	127	111	19	92	0	19	92
	2011			289	45	244	3	29	0	0	0	0	0	0	0
	1997		14♂67♀were remove from area C	81	14	67	5	107	69	54	33	21	0	33	21
	1998			135	47	88	4	81	64	42	23	19	0	23	19
	1999			177	70	107	7	128	0	0	0	0	0	0	0
	2000			177	70	107	0	0	0	0	0	0	0	0	0
C1	2001	80 individuals (15♂65♀) were remove to C2		97	55	42	5	44	36	23	14	9	0	14	9
	2002			120	69	51	0	0	0	0	0	0	0	0	0
	2003	64 individuals (39♂25♀) were remove to C4	13 mature individuals (6♂7♀) were introduce from Anhui nature reserve	69	36	33	10	174	111	75	33	42	0	33	42
	2004			144	69	75	4	52	0	0	0	0	0	0	0
	2005			144	69	75	3	52	0	0	0	0	0	0	0

	2006	29 individuals (29♂) were remove to C6		115	40	75	13	255	193	170	28	142	0	NC	NC
	2007			285	68	217	6	48	0	0	0	0	0	0	0
	2008			285	68	217	2	5	0	0	0	0	0	0	0
	2009			285	68	217	0	0	0	0	0	0	0	0	0
	2010	120 individuals (30♂90♀) were remove to C6		165	38	127	5	90	58	45	31	14	0	31	14
	2011			210	69	141	1	23	0	0	0	0	0	0	0
C2	2001		15♂65♀were introduced from area C; 15♂65♀were introduced from area C1	160	30	130	17	238	199	114	34	80	0	34	80
	2002			274	64	210	2	7	0	0	0	0	0	0	0
	2003	174 individuals (44♂130♀) were remove to C5	13 mature individuals (6♂7♀) were introduced from Anhui nature reserve	113	26	87	16	334	203	137	39	98	0	39	98
	2004			250	65	185	1	21	0	0	0	0	0	0	0
	2005			250	65	185	1	9	0	0	0	0	0	0	0
	2006	65 individuals (25♂40♀) were remove to C6		185	40	145	18	141	58	46	25	21	0	NC	NC
	2007			231	65	166	2	14	0	0	0	0	1♂1♀ born in 2006	0	0
	2008			229	64	165	2	14	0	0	0	0	0	0	0
	2009	80 individuals (20♂60♀) were remove to C6		229	64	165	1	4	0	0	0	0	0	0	0
	2010			149	44	105	6	103	51	44	22	22	0	22	22
	2011			193	66	127	1	16	0	0	0	0	0	0	0
C3	2003		162 individuals (30♂132♀) were introcued from C	175	36	139	5	107	61	45	34	11	0	34	11
	2004			220	70	150	5	64	0	0	0	0	0	0	0
	2005			220	70	150	5	33	0	0	0	0	0	0	0
	2006	120 individuals (30♂90♀) were remove to C6		100	40	60	11	207	101	89	29	60	0	NC	NC
	2007			189	69	120	5	33	0	0	0	0	1♂ born in 2006	0	0
	2008			188	68	120	1	17	0	0	0	0	0	0	0
	2009			188	68	120	3	24	0	0	0	0	0	0	0
	2010	90 individuals (30♂60♀) were remove to C6		98	38	60	4	71	40	37	15	22	0	15	22
	2011			135	53	82	5	102	46	41	6	35	0	6	35

C4	2003	64 individuals (39♂25♀) were introduced from C1;13 mature individuals (6♂7♀) were introduce from Anhui nature reserve	77	45	32	15	308	222	189	27	162	0	27	162
	2004		266	72	194	8	144	82	76	23	53	0	NC	NC
	2005		342	95	247	3	17	0	0	0	0	0	0	0
	2006	210 individuals (35♂175♀) were remove to C6	132	60	72	16	289	154	137	36	101	0	NC	NC
	2007		269	96	173	8	75	0	0	0	0	0	0	0
	2008		269	96	173	5	55	0	0	0	0	0	0	0
	2009		269	96	173	0	0	0	0	0	0	0	0	0
	2010	90 individuals (30♂60♀) were remove to C6	179	66	113	5	95	42	37	30	7	0	30	7
	2011		216	96	120	2	32	0	0	0	0	0	0	0
C5	2003	174 individuals (44♂130♀) were introduced from C2; 15 mature individuals (7♂8♀) were introduce from Anhui nature reserve	189	51	138	15	308	222	108	37	71	0	37	71
	2004		297	88	209	10	204	138	119	29	90	0	NC	NC
	2005		416	117	299	0	0	0	0	0	0	0	0	0
	2006	259 individuals (37♂222♀) were remove to C6	157	80	77	20	377	187	163	38	125	0	NC	NC
	2007		320	118	202	11	53	0	0	0	0	0	0	0
	2008		320	118	202	3	20	0	0	0	0	0	0	0
	2009		320	118	202	6	118	0	0	0	0	0	0	0
	2010	120 individuals (40♂80♀) were remove to C6	200	78	122	6	112	64	52	38	14	0	38	14
	2011		252	116	136	6	127	0	0	0	0	0	0	0
C6	2006	766 individuals (171♂595♀) were introduced from area C, C1, C2, C3, C4, C5	766	171	595	35	692	559	486	97	389	0	NC	NC
	2007		1252	268	984	31	568	443	401	101	300	1184 (301♂883♀)	NC	NC
	2008		469	68	401	15	276	144	99	43	56	0	NC	NC
	2009		568	111	457	6	144	47	38	24	14	0	NC	NC
	2010		1186	305	881	4	51	23	19	4	15	0	NC	NC
	2011		1205	309	896	13	251	84	69	31	38	0	NC	NC

Supplementary table S2

The calculated sex-age structure of the Chinese alligator (*Alligator sinensis)* sub-population in areas C and C1

The data in the table represent the number of individuals.

Area	Year	Total	Male					Female				
			<=2	3~6	7~9	>=10	Total	<=2	3~7	8~10	>=11	Total
C	1984	11	0	0	0	3	3	0	0	0	8	8
	1985	21	2	0	0	3	5	8	0	0	8	16
	1986	41	4	0	0	3	7	26	0	0	8	34
	1987	142	19	2	0	3	24	103	7	0	8	118
	1988	81	7	4	0	3	14	33	26	0	8	67
	1989	129	9	10	0	3	22	44	55	0	8	107
	1990	197	20	11	0	3	34	96	59	0	8	163
	1991	235	19	17	2	3	41	87	99	0	8	194
	1992	233	6	27	4	5	42	38	145	0	8	191
	1993	244	5	25	10	5	45	37	138	16	8	199
	1994	242	4	25	9	7	45	18	128	45	6	197
	1995	241	0	21	15	9	45	0	142	49	5	196
	1996	241	0	9	21	15	45	0	102	73	21	196
	1997	160	0	5	18	8	31	0	46	58	25	129
	1998	243	15	4	15	12	46	68	37	65	27	197
	1999	243	15	0	9	22	46	68	18	64	47	197
	2000	243	0	15	5	26	46	0	68	46	83	197
	2001	163	0	15	4	12	31	0	68	37	27	132
	2002	237	13	15	0	16	44	61	68	18	46	193
	2003	75	3	3	0	8	14	11	18	0	32	61
	2004	247	31	3	3	8	45	141	29	0	32	202
	2005	247	31	3	3	8	45	141	11	18	32	202
	2006	164	0	19	3	8	30	0	106	8	20	134
	2007	261	16	19	0	11	46	81	106	8	20	215
	2008	258	16	16	3	11	46	78	106	0	28	212
	2009	258	0	32	3	11	46	0	173	11	28	212
	2010	178	0	6	9	8	26	0	113	11	28	152
	2011	289	19	6	6	11	45	92	48	76	23	244

	1997	81	0	0	6	8	14	0	0	42	25	67
	1998	135	33	0	2	12	47	21	0	40	27	88
	1999	177	56	0	0	14	70	40	0	20	47	107
	2000	177	23	33	0	14	70	19	21	0	67	107
	2001	97	0	41	0	14	55	0	0	0	42	42
	2002	120	14	41	0	14	69	9	0	0	42	51
	2003	69	14	9	0	13	36	9	0	0	24	33
C1	2004	144	33	23	0	13	69	42	9	0	24	75
	2005	144	33	14	9	13	69	42	9	0	24	75
	2006	115	0	27	0	13	40	0	51	0	24	75
	2007	285	28	27	0	13	68	142	51	0	24	217
	2008	285	28	27	0	13	68	142	51	0	24	217
	2009	285	0	55	0	13	68	0	184	9	24	217
	2010	165	0	25	0	13	38	0	103	0	24	127
	2011	210	31	25	0	13	69	14	103	0	24	141
